# Differential recovery of chain-elongating bacteria: comparing droplet, plating, and dilution-to-extinction methods

**DOI:** 10.1128/msystems.01356-25

**Published:** 2025-10-30

**Authors:** Wannes Nauwynck, Myrsini Sakarika, Karoline Faust, Nico Boon

**Affiliations:** 1 Center for Microbial Ecology and Technology (CMET), Department of Biotechnology, Ghent University, Gent, Belgium; 2 Laboratory of Molecular Bacteriology, Rega Institute for Medical Research, Department of Microbiology, Immunology and Transplantation, KU Leuven, Leuven, Belgium; 3 Center for Advanced Process Technology for Urban Resource recovery (CAPTURE), Gent, Belgium; Universidad Miguel Hernandez de Elche, San Juan de Alicante, Alicante, Spain

**Keywords:** thermophilic microbiome, chain elongation, caproic acid, cultivation bias, droplet microfluidics, cultivability, rare biosphere, double emulsion

## Abstract

Microbial chain elongation via reverse β-oxidation offers a more sustainable route to produce medium-chain fatty acids like caproate, commodity chemicals typically produced via (petro)chemical processes. Thermophilic anaerobic microbiomes allow production at a high rate and selectivity but remain poorly understood due to the limited cultivability of their members. To better access functional taxa from a thermophilic chain-elongating reactor community, we applied multiple isolation strategies: conventional anaerobic plating, dilution-to-extinction (DTE), droplet-based microfluidics, and fluorescence-activated cell sorting (FACS). We evaluated the taxonomic range and cultivation success of each method using 16S rRNA gene sequencing. Each method yielded a distinct subset of microbial taxa. While *Clostridium acetireducens*-related strains were consistently isolated across all strategies, key thermophilic chain elongators (e.g., *Thermocaproicibacter melissae*-like organisms) only appeared in DTE. Droplet microfluidics enriched the most unique taxa in total, mostly rare taxa, including *Caproicibacter* and *Thermoanaerobacterium* spp. Plating yielded the lowest diversity, recovering only dominant taxa. FACS-based approaches failed to yield isolates, likely due to stress during processing. Comparing droplet-based isolation to DTE revealed critical insights: although droplets offer higher throughput, which intrinsically increases the chance of capturing rare taxa, not all DTE-cultivated organisms grew in droplets. This suggests additional contributing factors (apart from an increased throughput), such as encapsulation stress and droplet-specific microenvironments. These findings clarify the advantages and limitations of droplet cultivation strategies, allowing a more informed application of these techniques to access the so-called “microbial dark matter.”

**IMPORTANCE:**

Many environmentally and industrially relevant microbes remain uncultured, limiting our ability to understand and use them. This is especially true in thermophilic anaerobic microbiomes, which are promising systems for producing sustainable chemicals from organic waste streams. In this study, we explored how different cultivation strategies influence which microbes can be isolated from a thermophilic chain-elongating reactor. By comparing traditional and novel methods, including droplet microfluidics, we showed that each method recovers a unique set of microbes. While droplet-based methods enable high sampling depth with minimal effort and excel at isolating rare microbes, we found that they also introduce clear biases, as certain organisms recovered by other methods did not grow in droplets. Our work highlights the importance of the cultivation method in isolation success and helps shine a light on the selective forces at play in droplet-based microbial isolation.

## OBSERVATION

**Fig 1:**
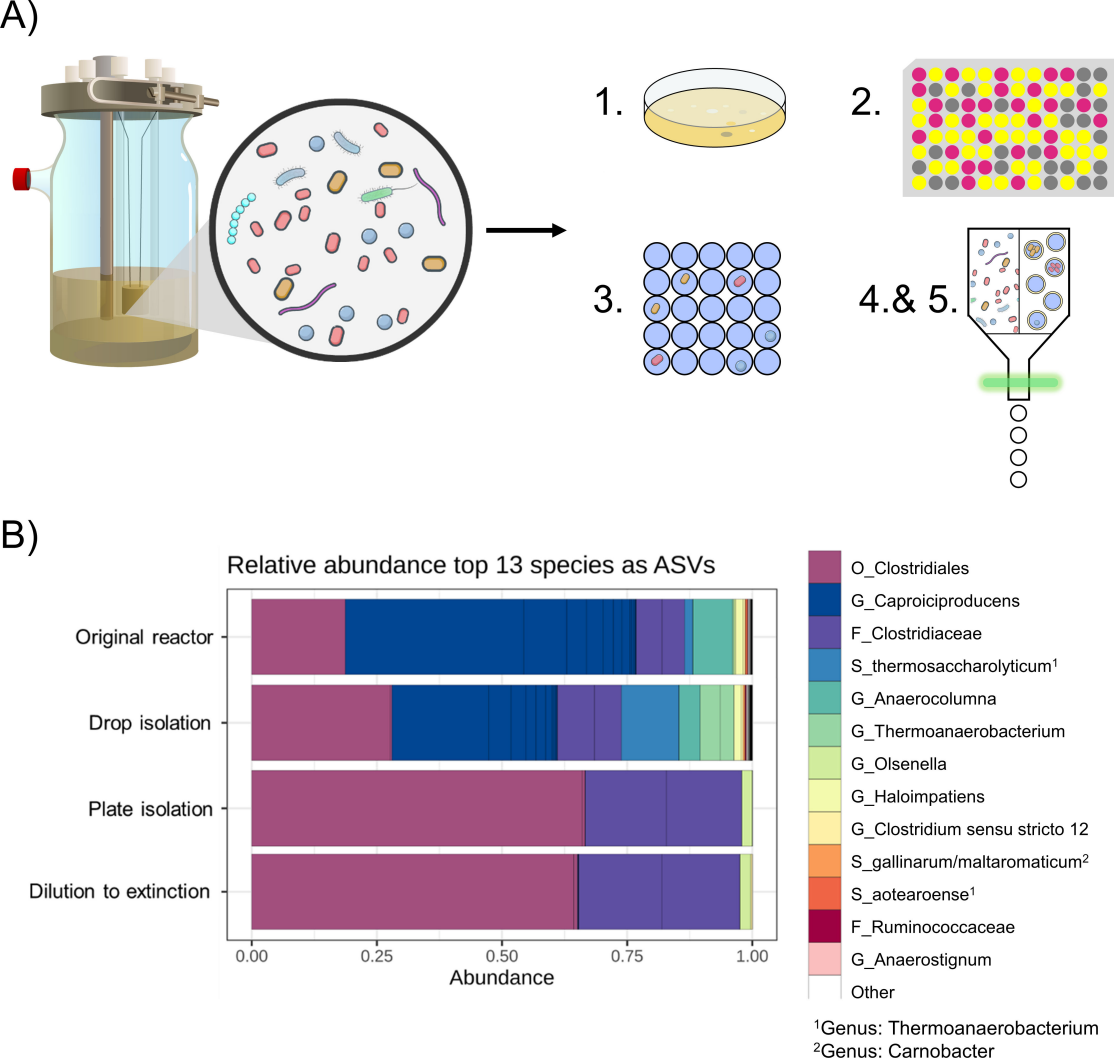
(**A**) Schematic of the thermophilic anaerobic chain-elongating reactor and the isolation process. A sample from the reactor was taken, and five different isolation methods were used to isolate a broad set of taxa: 1, conventional plating; 2, DTE; 3, droplet cultivation; 4, single-cell FACS; 5, double emulsion FACS. For method 5, droplets from the same cultivation pool as in method 3 were converted into double emulsions via vortexing and then screened by FACS. Only droplets showing biomass-associated fluorescence (SYBR Green signal) were sorted for downstream analysis. (**B**) Relative abundance of amplicon sequence variants (ASVs) of the top 13 species observed in plate isolation, droplet isolation, the original reactor, and in DTE. Names represent the closest taxonomic matches based on SILVA classification. (G: genus, S: species, F: family, O: order). Plate isolation and DTE show community profiles distinct from the original reactor, reflecting the outcome of extensive growth, starting from single cells to high biomass levels.

Reverse β-oxidation enables microbial chain elongation for the sustainable production of medium-chain fatty acids like caproate. This process is driven by anaerobic microbiomes, with thermophilic conditions potentially enhancing product titers (1). In a thermophilic chain-elongating reactor, we observed a highly diverse community dominated by mostly uncharacterized taxa, including putative caproate producers. To access and study these organisms, we aimed to recover as broad and representative a set of isolates as possible, using multiple isolation strategies. In parallel, we wanted to evaluate how these different isolation strategies influence overall cultivable diversity and the recovery of chain-elongating bacteria, which are often difficult to isolate (Fig. 1A) (2). Specifically, we compared conventional anaerobic plating, dilution-to-extinction (DTE) (3]), fluorescence-activated cell sorting (FACS), and droplet-based microfluidic cultivation (4) for their effectiveness in isolating functional key taxa from this thermophilic microbiome. Lastly, given the often-cited advantages of droplet cultivation but its mechanistic opacity (5), we used DTE—its conceptually most similar counterpart—as a reference to better understand the factors contributing to droplet isolation performance and to shed light on the “black box” of microbial isolation using droplet systems.

### Reactor community composition

We taxonomically profiled the reactor microbiome using 16S rRNA gene amplicon sequencing (V3–V4 region; Supplemental Information; Fig. 1B), identifying amplicon sequencing variants (ASVs) and their relative abundances. This reactor was operated at a hydraulic retention time of 4 days, at a pH of 6, and fed with an organic xylose-containing medium (Supplemental Information). The community was dominated by uncharacterized taxa, so we inferred potential metabolic functions based on the closest cultivated relatives.

The most abundant group (ASV4, ASV5, ASV9; ~48% of reads; ~96% identity) was closely related to *Thermocaproicibacter melissae*, a recently isolated thermophilic chain elongator (6). A second major group (ASV1–3; ~28%; ~96%) aligned with *Clostridium acetireducens*, an amino acid-degrading bacterium that reduces acetate to butyrate and isovalerate (7). Less abundant ASVs included saccharolytic fermenters related to *Anaerocolumna chitinilytica* (~8%; 98%) and *Thermoanaerobacterium* spp. (~2%; 99.8–100%), which likely metabolized xylose and biomass lysates potentially accumulating due to the reactor’s 4-day-long hydraulic retention time (8, 9).

### Isolating reactor members

To capture a diverse range of bacteria, we applied several cultivation strategies, including conventional plating, DTE, and droplet-based cultivation. We also explored less common techniques such as single-cell and double emulsion sorting (Fig. 1A). All were performed anaerobically using the same xylose-based medium fed to the reactor, differing only in the presence of reducing agents, to minimize potential oxygen exposure (Supplemental Information). In both sorting approaches, cells or droplets were stained with SYBR Green for biomass detection. Single-cell sorting deposits individual stained cells directly into wells, while double emulsion sorting transfers grown droplets into 96-well plates for further culturing (Supplemental Information). Neither sorting technique yielded isolates in our case, likely due to a combination of FACS-induced stress, transient oxygen exposure during FACS, or SYBR Green-induced stress.

### Cultivation methods yield distinct subsets of microbial taxa

**Fig 2:**
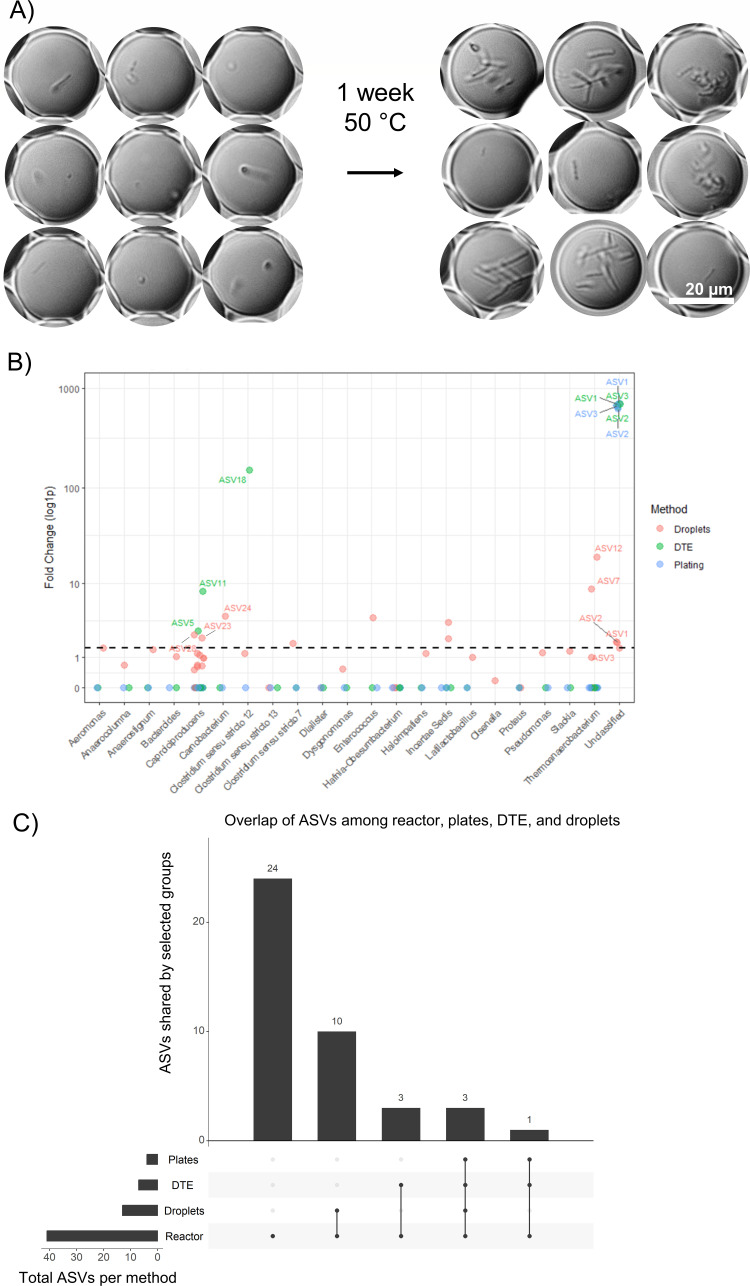
(**A**) Microbial growth in droplets. White scale bar: 20 µm. Droplet volume: 9 pL (26 µm diameter). Representative differential interference contrast microscopy images of droplets before and after incubation. Before incubation, only founder cells are visible; after incubation, droplets containing visible microcolonies indicate growth, while other founder cells do not grow. (**B**) Fold change in absolute abundance (*y*-axis, log1p transformed) from start to end of incubation for the most abundant genera in the reactor, across three isolation methods. A log1p scale [log(1  +  *x*)] is used to accommodate zero values and allow comparison across low- and high-abundance taxa. The horizontal line marks a 1.5-fold change in abundance. Each point represents a single ASV under one isolation method. Points are colored by method: red (droplet), green (dilution-to-extinction [DTE]), blue (plate). ASVs are grouped by genus, “Incertae sedis” and “Unclassified” are members of the family *Ruminococcaceae* and the order Clostridiales. (**C**) UpSet plot showing the distribution of unique and shared ASVs among the reactor and the used isolation methods: the original reactor, plate isolation, DTE, and droplet cultivation. Each vertical bar represents the number of ASVs found in the specific combination of sources indicated by the connected dots on the bottom of the graph.

Comparison across methods revealed that ASV1, ASV2, and ASV3*—C. acetireducens*-related strains (Supplemental Information)—were successfully cultivated by all three methods, indicating robust growth under the applied conditions (Fig. 2A and B). In contrast, ASV4, the most abundant *T. melissae*-related strain and likely a key caproate producer, was not recovered by any method. This may reflect specific growth requirements, stress sensitivity, or possibly syntrophic interactions (10).

Droplet cultivation specifically enriched several additional abundant ASVs (Fig. 2A through C), including *Thermoanaerobacterium* spp. (ASV7, ASV12), and successfully amplified rare taxa such as *Carnobacterium* (ASV24) and two *Caproicibacter* strains (ASV23, ASV28). Droplet cultivation notably did not cultivate *Olsenella* (ASV8), which was isolated by both plating and DTE. DTE recovered all taxa cultivated by plating and additionally enriched ASV5 and ASV11 (both likely caproate producers, Supplemental Information), and ASV18 (*Clostridium*), with similar throughput (Table S1), demonstrating broader cultivation success compared to plating and a differential cultivation ability compared to droplet cultivation.

Plating yielded the lowest richness among the tested methods, with no uniquely isolated taxa (Fig. 2C). While its lower throughput compared to droplet microfluidics partly explains this, it also underperformed relative to DTE, which had similar throughput (Table S1). This suggests additional biases. Plates allow interaction between colonies. This means that fast-growing taxa can outcompete others, with extended incubation leading to nutrient depletion, pH shifts by one strain that disadvantage others, and accumulation of inhibitory compounds (10–13). Colony formation also depends on biofilm formation, which may not be favored in planktonic reactor communities (14). Since all plated isolates (ASV1–3) were among the most abundant reactor members, competitive exclusion likely limited diversity recovery (15).

### Droplet cultivation outcome compared to DTE reveals effects beyond increased throughput

Droplet-based isolation is reported to capture a broader microbial diversity than plating (5, 16–18). However, the mechanisms remain unclear due to fundamental differences in spatial structure and competition between droplets and plate methods. In contrast, DTE and droplet microfluidics are conceptually similar, since both isolate single cells in physically separated, gas-permeable (microenvironments, yet have not been directly compared. This comparison is informative for understanding whether droplet microfluidics is simply a high-throughput extension of DTE, or if the difference in format (picoliter droplets vs. microliter wells) creates distinct outcomes. We identify three key factors potentially driving differences: increased throughput, encapsulation stress, and droplet-specific microenvironments.

The first key difference is throughput: droplet cultivation screens millions of droplets (100  µL of 9 pL droplets) compared to only hundreds of wells in DTE (7 × 96 wells total, with on average 1 cell/well, 443 grown wells), increasing the chance of recovering rare taxa for a comparable amount of effort (19). Indeed, our analysis highlight that droplet cultivation particularly favors rare taxa (Fig. S5). While DTE primarily cultivated taxa abundant or moderately abundant in the original reactor community, droplet cultivation uniquely enriched taxa present at lower initial abundances (0.01%–0.1%). Specifically, five rare taxa that were undetectable after DTE showed growth exclusively in droplets (Fig. S5), reinforcing prior observations that droplet microfluidics easily accesses rare microbes (16, 17).

However, if increased throughput were the sole reason behind differential cultivation in droplets, all taxa isolated via DTE should also appear in droplet isolates. This is not the case: *Olsenella* and several abundant *Caproiciproducens* ASVs were recovered only through DTE and did not grow in droplets. This lack of overlap strongly suggests that additional factors influence isolation success in droplet systems. We posit two main contributors. Firstly, the encapsulation process itself introduces shear stress, with our system staying below stress thresholds for *Escherichia coli* but surpassing them for other prokaryotes (Table S3), favoring organisms that are more tolerant to such conditions. Secondly, the microenvironment within droplets—characterized by small dimensions (~25 µm), rapid substrate depletion and product accumulation, and compound-dependent cross-talk between droplets—creates selective pressures distinct from microliter cultures in microwells (18, 20). These microenvironmental conditions remain difficult to characterize directly, making them a persistent “black box” in our understanding of droplet-based cultivation.

In summary, we demonstrate the recovery of previously uncharacterized organisms, highlight the role of cultivation method in shaping isolate recovery, and show that droplet-based cultivation enhances recovery of rare taxa, with possible additional contributions from encapsulation-induced stress and microenvironmental effects.

## SUPPLEMENTAL MATERIAL

Supplemental Information msystems.01356-25-s0001.pdf Additional photos of isolation outputs, extended results, and detailed materials and methods.

ASM does not own the copyrights to Supplemental Material that may be linked to, or accessed through, an article. The authors have granted ASM a non-exclusive, world-wide license to publish the Supplemental Material files. Please contact the corresponding author directly for reuse.
