# Supplementary material for: Differential recovery of chain-elongating bacteria: comparing droplet, plating, and dilution-to-extinction methods
Source: mSystems. 2025 Oct 30;10(11):e01356-25. doi: 10.1128/msystems.01356-25 (PMC12625695; doi:10.1128/msystems.01356-25)
Supplement: Supplemental Information — Additional photos of isolation outputs, extended results, and detailed materials and methods. [file msystems.01356-25-s0001.pdf]

Supplementary information: Differential recovery of chain-elongating bacteria: comparing droplet, plating, and dilution-to-extinction methods

Wannes Nauwynck[1,2,3], Myrsini Sakarika[1,3], Karoline Faust[2] & Nico Boon[1,3]*

[1] Center for Microbial Ecology and Technology (CMET), Department of Biotechnology, Ghent University – Coupure Links 653, 9000, Ghent, Belgium

[2] Laboratory of Molecular Bacteriology (Rega Institute), Department of Microbiology, Immunology and Transplantation, KU Leuven, – Herestraat 49, 3000, Leuven, Belgium

[3] Center for Advanced Process Technology for Urban Resource recovery (CAPTURE), Frieda Saeysstraat 1, 9000 Gent, Belgium

Acronyms:

ASV: amplicon sequence variant

DTE: dilution-to-extinction

DE: double emulsion

FACS: fluorescence-activated cell sorting

PBS: phosphate-buffered saline

Supplementary results

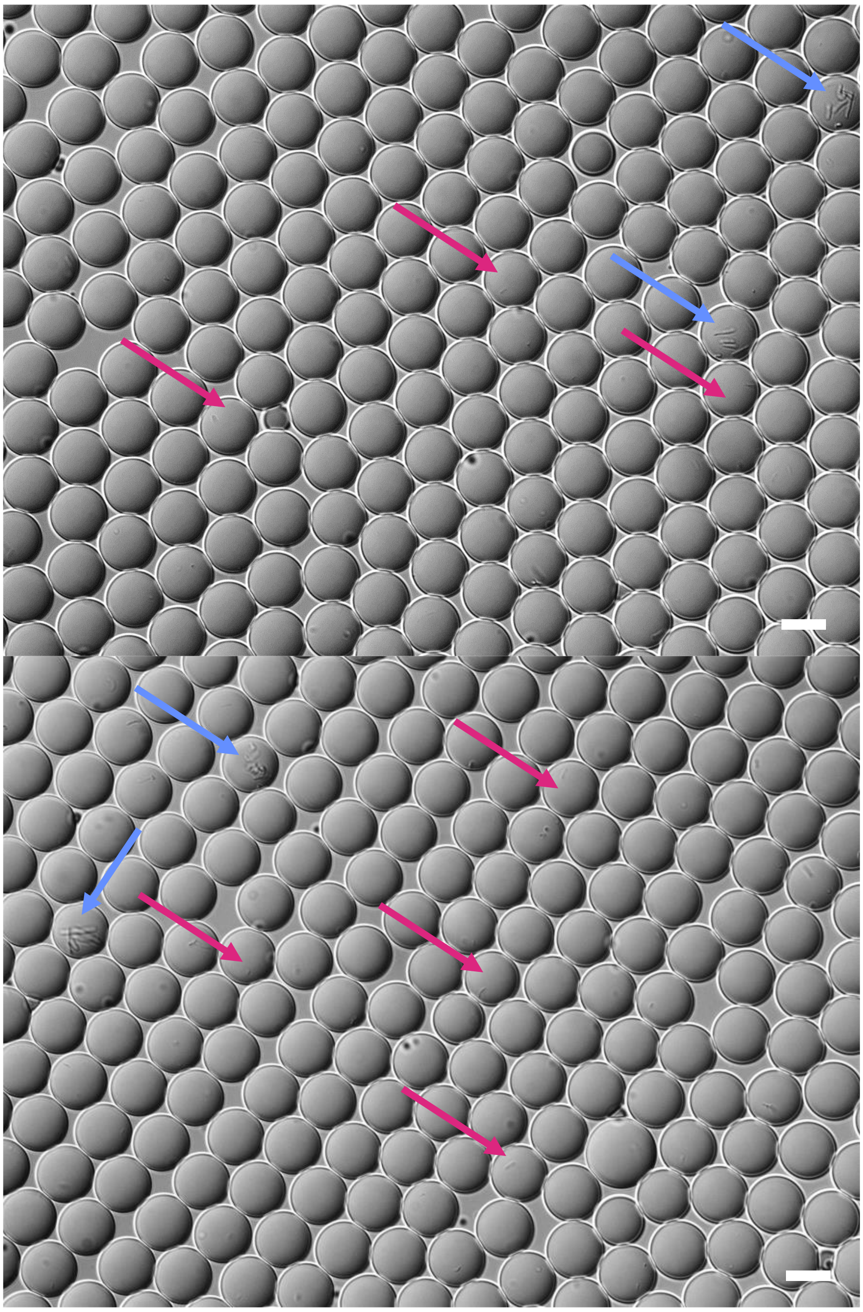

Fig. S 1 Microscopy images of droplet cultivation at the end of the incubation period. White scale bar: 20 µm; droplet volume: 9 pL (26 µm diameter). Most droplets appear empty, with some containing a single ungrown founder cell (magenta arrow) and a smaller fraction showing successful growth into microcolonies (blue arrow).

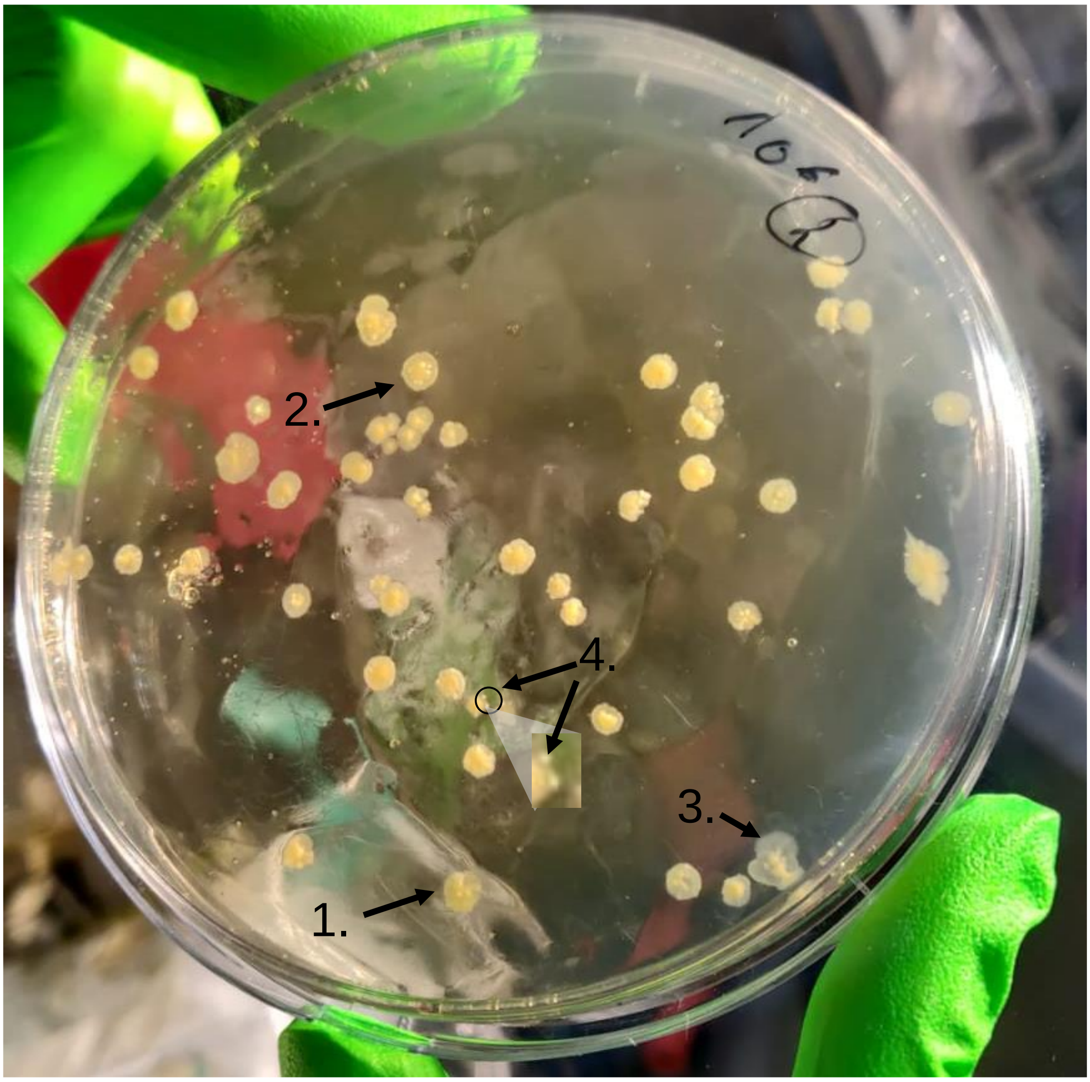

Fig. S 2 Thermophilic anaerobic enrichment plated on solid medium showing different colony morphologies. 1. Moderate sized yellow, round colonies with entire margin, 2. Moderate sized pale pink, round colonies, with entire margin, 3. Moderate to large pale pink irregular colonies with undulate margin, 4. White round microcolonies adjacent to moderate-sized colony.

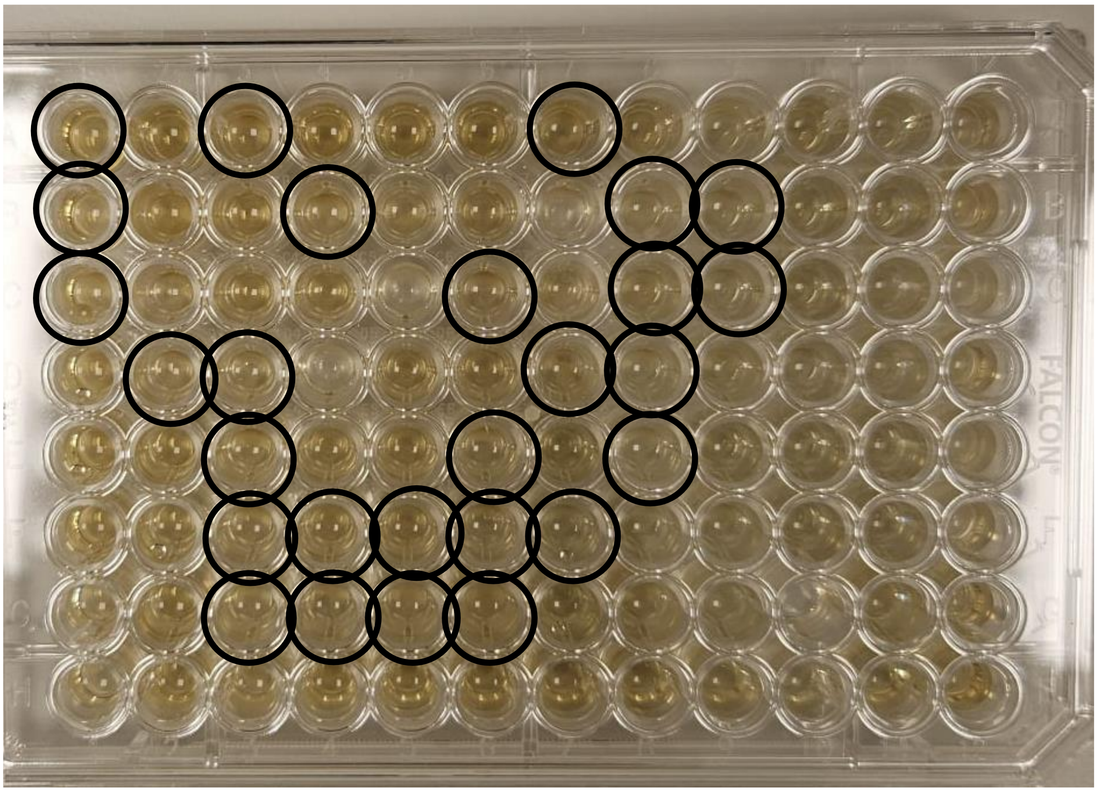

Fig. S 3 96-well plate from dilution-to-extinction isolation from thermophilic anaerobic enrichment grown under thermophilic anaerobic conditions (10^{-10} dilution shown). Wells with visible biomass, observed as sedimented cell pellets at the bottom of the well, are marked with black circles

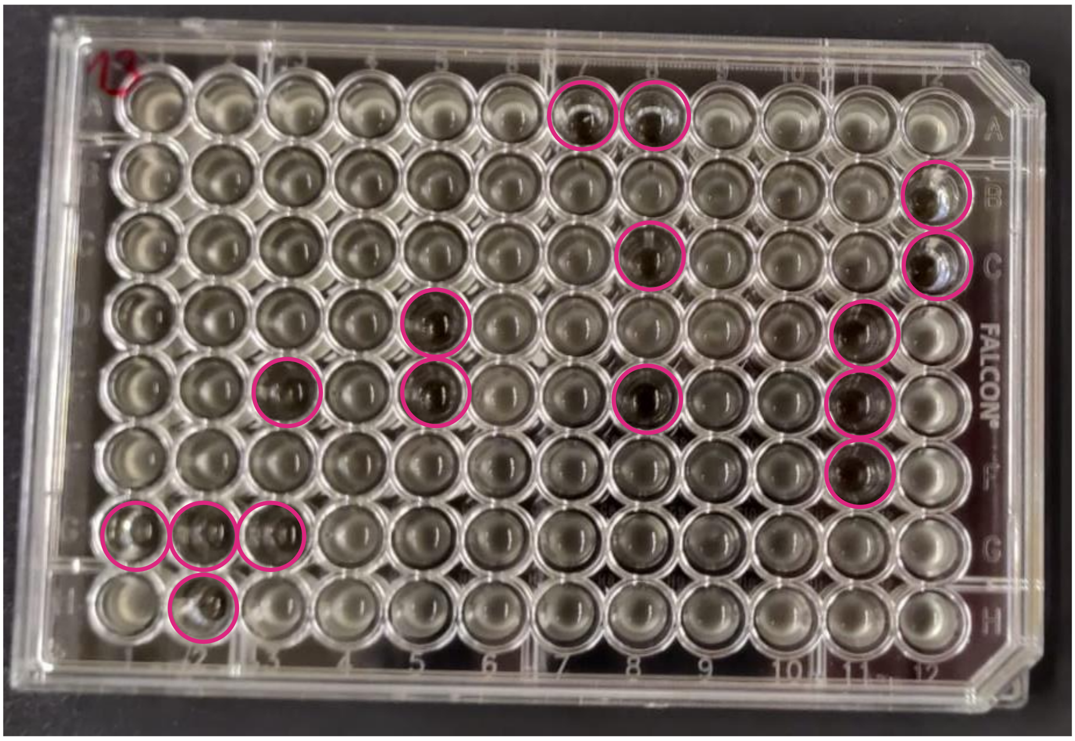

Fig. S 4 Single E. coli cells were sorted into a 96-well plate containing LB broth. After overnight incubation, robust growth was observed in nearly all wells, indicating successful clonal outgrowth from individual cells. Wells without growth are indicated with red circles.

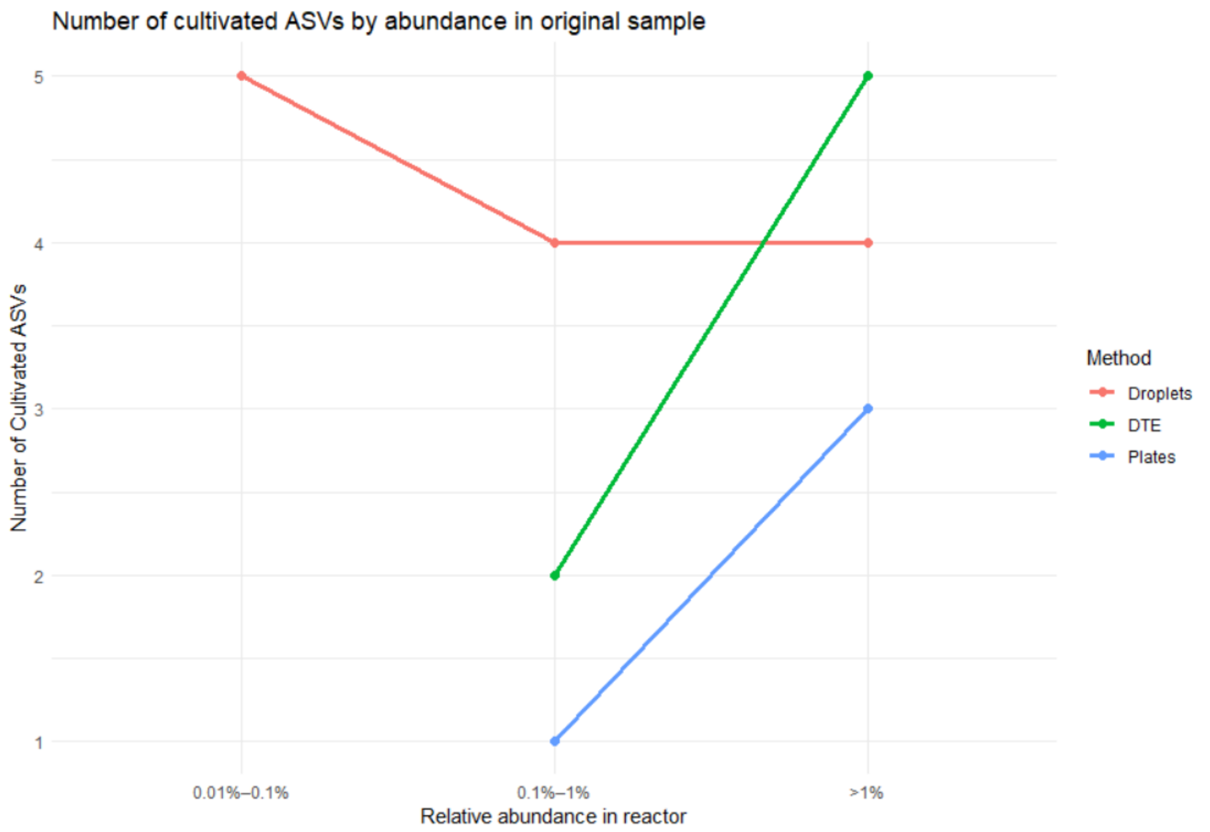

Fig. S 5. Number of ASVs successfully cultivated by each method (droplets, DTE, and plating), grouped by their relative abundance in the original reactor sample (t_0). ASVs were binned into three abundance categories, and cultivation was defined as a >1.5-fold increase in absolute abundance. The plot highlights that droplet-based cultivation consistently recovers more ASVs from lower-abundance bins compared to other methods, consistent with its higher throughput.

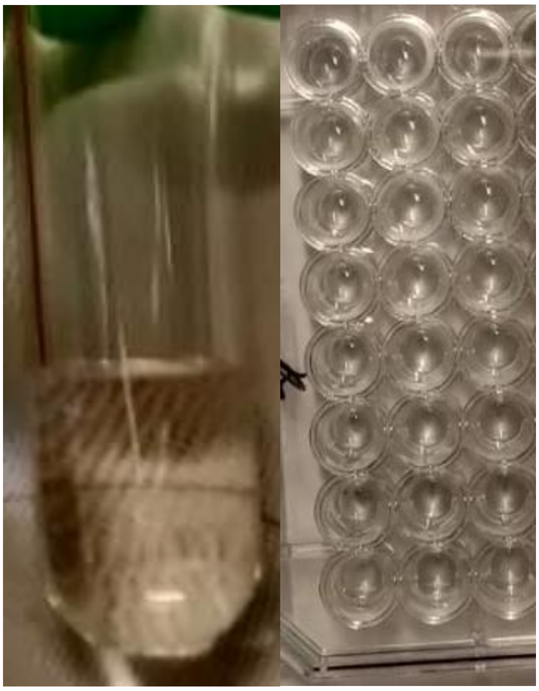

Fig. S 6. Anaerobicity of samples during sorting. Left: FACS tube containing stained cells remained transparent throughout the entire sorting procedure, indicating maintenance of anaerobic conditions. Right: Wells of the 96-well plate filled with medium also remained transparent during sorting, further confirming anaerobic conditions were preserved.

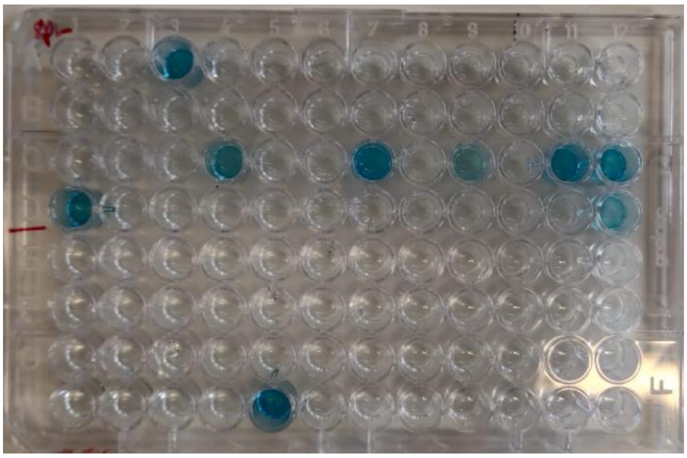

Fig. S 7 FACS accuracy control using HRP-filled double emulsions sorted into TMB-containing wells. The top four rows represent sorted HRP-positive double emulsions, and the bottom four rows represent sorted background (noise) events. A total of 8 out of 48 wells in the target fraction turned blue, indicating successful deposition of HRP-containing droplets. The modest recovery reflects the known challenges of sorting double emulsions due to their large size

relative to the 100 µm nozzle. One well in the background fraction also turned blue, possibly due to accidental inclusion of a small double emulsion in the sorted blank fraction.

Table S 1 Estimated number of founder cells initially inoculated and sequenced for each cultivation method. Plate counts are based on 1 plate (50 µL of 10^{-6} dilution), dilution-to-extinction on 7 plates with 1 cell/well on average (10^{-10} dilution), and droplets on 100 µL of 9 pL droplets with 4% aimed occupancy.

**Method**	**Amount of founder cells sequenced**
Plates	500
Dilution-to-extinction	672
Droplets	300 000

Table S 2 Most abundant amplicon sequence variants (ASVs) detected in the thermophilic chain-elongating reactor. Columns indicate: (1) ASV identifier, (2) relative abundance in the reactor community, (3) taxonomic classification based on the SILVA database, (4) closest cultivated match based on NCBI BLAST results, (5) percentage identity of the 16S rRNA gene sequence to the closest cultivated match, and (6) source information for the cultivated reference organism.

	**Relative percentage of ASVs in reactor (%)**	**Silva**	**BLAST**	**BLAST match (%)**	**Reference**
ASV1	18,68	/	*Clostridium acetireducens* 30A	96,27	(1)
ASV2	5,18	/	*Clostridium acetireducens* 30A	96.02	(1)
ASV3	4,45	/	*Clostridium acetireducens* 30A	96.02	(1)
ASV4	35,68	*Caproiciproducens*	*Thermocaproicibacter melissae strain* MDTJ8	99,75	(2)
ASV5	8,56	*Caproiciproducens*	*Thermocaproicibacter melissae strain* MDTJ8	100	(2)
ASV6	7,91	*Anaerocolumna*	*Anaerocolumna chitinilytica*	98,27	(3)
ASV7	1,66	*Thermoanaerobacterium*	*Thermoanaerobacterium thermosaccharolyticum strain* GD17	100	(4)
ASV8	0,33	*Olsenella*	/	/	/

ASV9	3,97	*Caproiciproducens*	*Thermocaproicibacter melissae strain* MDTJ8	99,25	(2)
ASV10	3,33	*Caproiciproducens*	*Caproicibacter glucoseutiliensis* BJN0012	97,76	(5)
ASV11	2,02	*Caproiciproducens*	*Caproicibacter glucoseutiliensis* BJN0012	98,26	(5)
ASV12	0,26	*Thermoanaerobacterium*	*Themoanaerobacterium calidifontis* Rx1	99,75	(6)
ASV13	1,71	*Caproiciproducens*	*Caproicibacter glucoseutiliensis* BJN0012	97,51	(5)
ASV14	1,51	*Haloimpatiens*	*Haloimpatiens lingqiaonensis* KCTC15321	100	(7)
ASV18	0,40	*Clostridium* sensu stricto 12	*Clostridium algifaecis strain* MB9-9	98,51	(8)
ASV24	0,11	*Carnobacterium*	*Carnobacterium maltaromaticum strain* MMF-31	100	(9)
ASV28	0,15	*Caproiciproducens*	*Oscillospiraceae bacterium strain* MG12	99	(10)
ASV29	0,10	Incertae Sedis	*Faecalispora Sphorosphaeroides or jeddahensis*	100	(11, 12)

Table S 3 Reported pressure sensitivities of selected strains

**Species**	**Shear/Pressure Threshold**	**Observed Effect**	**Reference**
Escherichia coli	Up to 1,250 Pa	No notable viability loss	(13)
E. coli	4,240 Pa (steady shear, prolonged exposure)	No measurable damage	(14)
Bacillus subtilis	1,482 s^{-1} (shear rate)	Oxidative stress, viability loss	(15)
Lactobacillus bulgaricus	72 Pa	Sublethal stress	(16)
Eubacterium ramulus	Mild shear (in small flasks)	Strong growth inhibition	(17)
Caproic acid microbial community	208.5 Pa, 2.09×10^4 s^{-1} (calculated using Navier-Stokes)	Below known *E. coli* thresholds; potentially stressful to sensitive species	This study

Supplementary materials and methods

Reactor operation

The sample was taken from thermophilic, anaerobic, chain elongating reactor fed with xylose-containing medium (see Xylose-based medium preparation for recipe), operated at a hydraulic retention time of 4 days and a working volume of 1 L. The reactor was continuously mixed with a magnetic stirrer (ProSense, Netherlands) at 300 rpm. The pH was kept constant at 6.0 using a pH controller (Verderflex, UK) and 4 M NaOH. The temperature was kept constant using a water bath at 50°C. To ensure the presence of relevant communities, 4 different samples of thermophilic anaerobic digesters were used as inoculum, which were initially inoculated at 10% v/v. The sample from which all isolation efforts were done was taken anaerobically the 100th day of reactor operation, when a desired product profile was obtained.

Xylose-based medium preparation

In this work, the xylose-based medium was used to support the reactor operation, and was used during all isolation efforts. For a 1-liter batch, inorganic salts were added via stock solutions: 10 mL of 100x KH_2PO_4 stock (78 g/L), 40 mL of 25x stock containing NH_4Cl (50 g/L), $MgCl_2 \cdot 6H_2O$ (50 g/L), $MgSO_4 \cdot 7H_2O$ (1.25 g/L), and $CaCl_2 \cdot 2H_2O$ (12.5 g/L). From a 40x organic nutrients stock solutions 25 mL was added. The stock solution contained yeast extract (40 g/L) and tryptone (40 g/L). Trace elements and vitamins were supplied from refrigerated 10× stock solutions (Se-W, SL-10, and 7-vit, each added at 1 mL per liter, see Mariën et al., 2022). Xylose (Carl Roth, Germany) was used as the sole carbon source, added from a 400 g/L stock (100 mL to reach 40 g/L final). To buffer the system, MES (2-(N-morpholino) ethanesulfonic acid, Merck, Germany) was added using 100 mL of a 1 M stock. For all isolations anaerobic conditions were maintained by adding following reducing agent stocks to a final concentration of 5 mM: cysteine HCl (285 mM stock, Merck, Germany) and thioglycolate (877 mM stock, Merck, Germany). Redox levels were monisstored by adding 0.5 mL of sodium resazurin (1 g/L stock, Merck,

Germany) as an indicator. Sterile water was used to bring the final volume to 1 L. All stock solutions were sterilized separately. When preparing solid media, 10 g of gellan gum (Merck, Germany) was included with the organic nutrients stock solution before sterilization.

Anaerobic medium preparation

Preparation of anaerobic solutions (demineralized water, xylose medium, 10x PBS) happened under identical conditions. Each solution was transferred into a Schott bottle and heated with intermittent shaking until boiling, which was maintained for approximately one minute to remove dissolved oxygen. While cooling in an ice bath, each solution was sparged with nitrogen gas for 30 minutes. After cooling, the bottles were sealed with a stopper and red ring cap, and the headspaces were flushed with nitrogen gas for 10-15 minutes until a slight overpressure (~0.1 bar) was reached. The solutions were autoclaved.

Cell counts

Cell concentrations of the reactor sample were determined using an Attune NxT BRXX flow cytometer (Thermo Fisher Scientific, USA) with SYBR Green I (Thermo Fisher Scientific, USA) staining. Bacterial samples were diluted 1,000-fold and 10,000-fold in 0.22 µm-filtered phosphate-buffered saline (PBS) prior to analysis. A 0.22 µm-filtered reactor sample was included to help distinguish between cellular and non-cellular background. Data acquisition was performed using an event threshold of 200 in the 530 ± 15 nm emission filter (488 nm excitation). A total of 100 µL per sample was aspirated at a flow rate of 100 µL/min. All dilutions were performed in technical triplicate and measured as such to ensure reproducibility.

Dilution-to-extinction

Tenfold serial dilutions were prepared in growth medium inside an anaerobic chamber, down to 10^{-8}. Further dilutions to 10^{-9} and 10^{-10} were made by adding 20 µL of the preceding dilution to 180 µL of growth medium into 96-well plates. The dilutions were informed by the cell density in the reactor sample, with both 10^{-9} and 10^{-10} averaging 10.8 cells per well and 1.08 cells per well on average. Microtiter plates were incubated in anaerobic jars at 50 °C and checked monthly for growth. Anaerobic jars were flushed each time with N_2:CO_2 (Air Liquide, France) after opening.

Plate isolation

Tenfold dilutions were made in growth medium in an anaerobic chamber. Plating was performed by spotting 50 µL of each dilution (10^{-6} to 10^{-10}) onto agar plates in duplicate and spreading the cells using a sterile cell spreader. The plates were incubated in anaerobic jars at 50 °C and checked monthly for growth. Anaerobic jars were flushed each time with N_2:CO_2 after opening.

Anaerobic droplet cultivation

Droplets were generated in an anaerobic chamber using microfluidic chips with a channel height of 10 µm. A bacterial cell suspension consisting of reactor sample resuspended in fresh growth medium until a concentration of $2.5*10^7$ cells/mL was flowed at 250 µL/hr, and a continuous phase of HFE7500 (3M, USA) + 1 wt% Picosurf (Sphere Fluidics, UK) was flowed at 400 µL/hr. Prior to droplet generation, the pumps, chip, oil and tubing were placed in an anaerobic chamber for equilibration with the oxygen-free atmosphere. Droplet formation was initiated without direct observation and continued until droplets emerged from the outlet tubing, followed by an additional 5-minute equilibration period. Droplets were then collected for one hour. The quality of the single emulsions was established using microscopy. After quality assurance, the collected droplets were subsequently injected into anaerobic Hungate tubes, which were modified with hydrophobic 2 mL tubes connected to neoprene tubing at the outlet to enable droplet sampling through the Hungate rubber stopper.

16S rRNA gene amplicon sequencing

Representative samples from the three successful isolation methods were collected for DNA extraction: 1 mL of the original reactor sample; colonies scraped from agar plates and pooled into a single tube; pooled cultures from DTE wells (20 µL each); and 100 µL of droplets coalesced using perfluoro-octanol. The first three sample types were pelleted by centrifugation (20.000xg ,1 min) and stored at −20 °C until DNA extraction. Genomic DNA was extracted using the DNeasy PowerSoil Pro Kit (Qiagen, Germany). A 10 µL aliquot of the extracted DNA was submitted to LGC Genomics GmbH (Berlin, Germany), where the V3-V4 region of the 16S rRNA gene was amplified using primers 341F and 785R. Amplicon libraries were prepared and sequenced on an Illumina MiSeq platform with v3 chemistry (Illumina, USA).

The DADA2 R package was used to process the amplicon sequence data following the pipeline described by (19). Primer sequences were first removed, and reads were truncated at a quality score threshold (truncQ=2). Additional filtering excluded reads with ambiguous bases or high expected errors (maxEE=2,2). After dereplication, reads were denoised using the Divisive Amplicon Denoising Algorithm (DADA) with the selfConsist sample inference method and pooling enabled. Error rates were inspected, and denoised reads were then merged. The resulting ASV table, following chimera removal, was used for taxonomic classification with the Naive Bayesian Classifier and the DADA2-formatted Silva v138 database (20).

While taxonomic characterization using 16S rRNA gene amplicon sequencing provides informative insights, several methodological limitations must be considered. First, assigning physiological functions based on related 16S sequences is inherently limited due to significant genomic variability and physiological differences among even closely related strains; thus, precise metabolic predictions cannot reliably be made solely from 16S data (21). Additionally, the reported relative abundances of ASVs can be biased by factors such as variable 16S gene copy numbers across taxa, primer biases, and amplification efficiency, potentially distorting the true microbial abundances (22).

FACS isolation

All manipulations prior to sorting were executed in an anaerobic cabinet. The sorter itself was in an aerobic environment but measures were taken to maintain anaerobicity of the sample. The reactor sample was adjusted to a concentration of 10^5-10^6 cells/mL using anaerobic PBS (with 5 mM HCl-cysteine and 5 mM sodium thioglycolate), ensuring sufficient event rates for high-throughput sorting while preserving single-cell resolution. To enhance post-sort viability and minimize DMSO toxicity, a highly concentrated 10,000× SYBR Green stock was used, of which 1 µL was added to 10 mL of cell suspension. Cells were stained with SYBR Green I for 20 minutes at 37 °C inside an anaerobic chamber. Stained samples were then transferred to the sorter in sealed anaerobic gas jars to maintain low-oxygen conditions.

Cell sorting was performed using a BD FACS Melody equipped with 488 nm (blue) and 561 nm (yellow-green) lasers (BD, USA). SYBR Green-positive events were gated based on a stained control sample (stained anaerobic PBS). Sorting was conducted in single-cell mode, depositing one cell per well into 96-well plates pre-filled with anaerobic growth medium. An event rate of 600-800 events per second was maintained throughout sorting.

To confirm sterility and sorting precision, several controls were included: (i) Unsorted media blanks to detect background contamination, (ii) “No sort” controls, where plates were handled identically but received no cells, (iii) “Blank sort” controls, where background noise events were sorted into wells. As a positive control, SYBR Green I-stained *E. coli* from an overnight exponential culture was sorted into LB medium (Miller). The majority of wells showed growth after overnight incubation, confirming both the viability of sorted cells and accuracy of the single-cell deposition (Fig. S 4).

Throughout sorting, anaerobic conditions in both the stained sample and the microtiterplates filled with medium were maintained, with the sorter in an aerobic environment. This was done as follows: stained sample and destination plates were briefly removed from the anaerobic jars (<5 minutes) for transfer to the sorter, then returned immediately after sorting. After which anaerobic jars were flushed with a N_2:CO_2 gas mixture to minimize oxygen exposure after

opening of the jars. All media included resazurin as a redox indicator; all cell suspensions and 96-well plates remained fully reduced (colorless) throughout the procedure (Fig. S 6). The sorted plates were incubated at 50 °C and inspected monthly, after each inspection the jars were flushed with N_2:CO_2 gas.

Double emulsion-FACS cultivation

To isolate and culture droplets containing grown bacteria, we developed an anaerobic FACS workflow for double emulsions (DEs). A sterile anaerobic DE buffer was prepared by combining an anaerobic filter sterilized 20 w% Tween20 stock with autoclaved 10x PBS to provide isotonic, physiological conditions and droplet stabilization during sorting. To maintain anaerobicity, 4.38 mL of cysteine-HCl (285 mM), 1.43 mL of sodium thioglycolate (877 mM), and 250 µL of resazurin (1 g/L) were added as reducing agents and redox indicator, respectively. The final volume was adjusted to 250 mL with sterile anaerobic water, resulting in a sterile anaerobic buffer containing 1.75 w% Tween20 + 2x PBS. DEs were made by vortexing as seen in (23). The vortexer used was a VWR Analog vortex mixer (VWR, USA) DEs were stained anaerobically using SYBR Green (1 µL of a 10,000× DMSO stock per 10 mL sample), followed by a 30-minute incubation at 37 °C. Samples were kept anaerobic throughout staining and handling. Sorting was performed on a BD FACS Melody (488 nm and 561 nm lasers), using unstained DEs for gating. To enrich for droplets with growth, the top 15% of SYBR Green-positive events were sorted into 96-well plates containing anaerobic media. All sorting was conducted using the same anaerobic sorting workflow with gas jars and aerobic sorter (as stated in FACS); six plates were sorted in total. Controls included media blanks (no events sorted, not opened), "no sort" sort plates (no events sorted, left open in the FACS machine as an indication of contamination of FACS aerosol spray), and background sorts of low-fluorescence events, as an indication of FACS sheath contamination. None of the negative control plates showed growth. To validate single-droplet sorting accuracy, DEs filled with horseradish peroxidase (5 mg/mL in PBS) were sorted into wells containing 3,3′,5,5′-Tetramethylbenzidine substrate. Wells turning blue confirmed correct deposition (Fig. S 7).

Microscopy

All single emulsion populations were imaged in a urine sediment counting chamber (Novolab, Belgium). First, the chamber was filled with HFE-7500, after this, about 1 µL single emulsions were added to the droplet chamber. The chamber was incubated for 2 minutes to allow a droplet monolayer to form. Pictures were taken with an Axioskop 2 plus (Zeiss, Germany) equipped with a Q-Imaging Retiga R3 CCD Camera (Cairn Research, UK).

Data analysis

To identify the taxa that grew, 16S rRNA amplicon sequencing was performed before and after incubation. Absolute abundances were calculated by scaling relative 16S abundances with flow cytometry-based total cell counts. For droplet cultivation the cells proliferated 1.26-fold over a week of growth (from $2.52 \pm 0.18 *10^7$ cells/mL to $3.18 \pm 0.25 *10^7$ cells/mL). For the plate isolation and dilution-to-extinction (DTE) samples, absolute cell counts were not determined, as relative abundances already reflect the composition of bacteria that successfully grew. This is because in these enrichment-based methods, non-growing cells are effectively diluted out, while growing organisms typically reach high densities ($\geq 10^8$-10^9 cells/mL). Therefore, the presence of an ASV in the 16S rRNA gene data from these samples is considered strong evidence of growth. However, to enable direct comparison with droplet-based absolute abundance estimates, a cell density of $5*10^9$ cells/mL was applied uniformly to these samples, derived from the reactor biomass cell counts and serving as a proxy of expected cell counts here. In all following analyses, we derive growth in from fold-growth in absolute abundance of an ASV (i.e. absolute abundance after incubation divided by absolute

abundance after before incubation), we use an arbitrary threshold of 1.5-fold amplification as an indication of growth.

References

1. Örlygsson J, Krooneman J, Collins MD, Pascual C, Gottschal JC. 1996. Clostridium acetireducens sp. nov., a novel amino acid-oxidizing, acetate-reducing anaerobic bacterium. Int J Syst Bacteriol 46:454–459.

2. Van Nguyen T, Viver T, Smets I, Bernaerts K, Faust K, Lavigne R, Poughon L, Dussap CG, Springael D. 2023. Thermocaproicibacter melissae gen. nov., sp. nov., a thermophilic chain-elongating bacterium, producing n-caproate from polymeric carbohydrates. Int J Syst Evol Microbiol 73:005893.

3. Ueki A, Tonouchi A, Kaku N, Ueki K. 2021. Anaerocolumna chitinilytica sp. nov., a chitin-decomposing anaerobic bacterium isolated from anoxic soil subjected to biological soil disinfestation. Int J Syst Evol Microbiol 71:004999.

4. Li T, Zhang C, Yang KL, He J. 2018. Unique genetic cassettes in a thermoanaerobacterium contribute to simultaneous conversion of cellulose and monosugars into butanol. Sci Adv 4:e1701475.

5. Dai M, Xu Y, Zhao L, Wu M, Ma H, Zhu L, Li W, Li X, Sun B. 2024. Caproicibacter sp. BJN0012, a potential new species isolated from cellar mud for caproic acid production from glucose. J Biotechnol 388:11–23.

6. Shang SM, Qian L, Zhang X, Li KZ, Chagan I. 2013. Themoanaerobacterium calidifontis sp. nov., a novel anaerobic, thermophilic, ethanol-producing bacterium from hot springs in China. Arch Microbiol 195:439–445.

7. Wu D, Zhang NF, Sun C, Zhang WW, Han SB, Pan J, Wu M, Dilbar T, Zhu XF. 2016. Haloimpatiens lingqiaonensis gen. Nov., sp. nov., An anaerobic bacterium isolated from paper-mill wastewater. Int J Syst Evol Microbiol 66:628–632.

8. Wu YF, Zheng H, Wu QL, Yang H, Liu SJ. 2014. Clostridium algifaecis sp. nov., An anaerobic bacterial species from decomposing algal scum. Int J Syst Evol Microbiol 64:3844–3848.

9. Loch TP, Kumar R, Xu W, Faisal M. 2011. Carnobacterium maltaromaticum infections in feral Oncorhynchus spp. (Family Salmonidae) in Michigan. J Microbiol 49:703–713.

10. Candry P, Chadwick GL, Caravajal-Arroyo JM, Lacoere T, Winkler MKH, Ganigué R, Orphan VJ, Rabaey K. 2023. Trophic interactions shape the spatial organization of medium-chain carboxylic acid producing granular biofilm communities. ISME J 17:2014–2022.

11. Lagier JC, Bibi F, Ramasamy D, Azhar EI, Robert C, Yasir M, Jiman-Fatani AA, Alshali KZ, Fournier PE, Raoult D. 2015. Non contiguous-finished genome sequence and description of Clostridium jeddahense sp. nov. Stand Genomic Sci 9:1003–1019.

12. Cibis KG, Gneipel A, König H. 2016. Isolation of acetic, propionic and butyric acid-forming bacteria from biogas plants. J Biotechnol 220:51–63.

13. Lange H, Taillandier P, Riba JP. 2001. Effect of high shear stress on microbial viability. J Chem Technol Biotechnol 76:501–505.

14. Vettori D, Manes C, Dalmazzo D, Ridolfi L. 2022. On Escherichia coli Resistance to Fluid Shear Stress and Its Significance for Water Disinfection. Water (Switzerland) 14:2637.

15. Sahoo S, Rao KK, Suraishkumar GK. 2006. Reactive oxygen species induced by shear stress mediate cell death in Bacillus subtilis. Biotechnol Bioeng 94:118–127.

16. Arnaud JP, Lacroix C, Foussereau C, Choplin L. 1993. Shear stress effects on growth

and activity of Lactobacillus delbrueckii subsp. bulgaricus. J Biotechnol 29:157–175.

17. Schmidt A. 2011. Biokatalytische Gewinnung von Flavonoiden.

18. Mariën Q, Ulčar B, Verleyen J, Vanthuyne B, Ganigué R. 2022. High-rate conversion of lactic acid-rich streams to caproic acid in a fermentative granular system. Bioresour Technol 355:127250.

19. Callahan BJ, McMurdie PJ, Rosen MJ, Han AW, Johnson AJA, Holmes SP. 2016. DADA2: High-resolution sample inference from Illumina amplicon data. Nat Methods 13:581–583.

20. Quast C, Pruesse E, Yilmaz P, Gerken J, Schweer T, Yarza P, Peplies J, Glöckner FO. 2013. The SILVA ribosomal RNA gene database project: Improved data processing and web-based tools. Nucleic Acids Res 41:D590–D596.

21. Matchado MS, Rühlemann M, Reitmeier S, Kacprowski T, Frost F, Haller D, Baumbach J, List M. 2024. On the limits of 16S rRNA gene-based metagenome prediction and functional profiling. Microb Genomics 10:1203.

22. Louca S, Doebeli M, Parfrey LW. 2018. Correcting for 16S rRNA gene copy numbers in microbiome surveys remains an unsolved problem. Microbiome 6:41.

23. Wang J, Hahn S, Amstad E, Vogel N. 2022. Tailored Double Emulsions Made Simple. Adv Mater 34:2107338.
